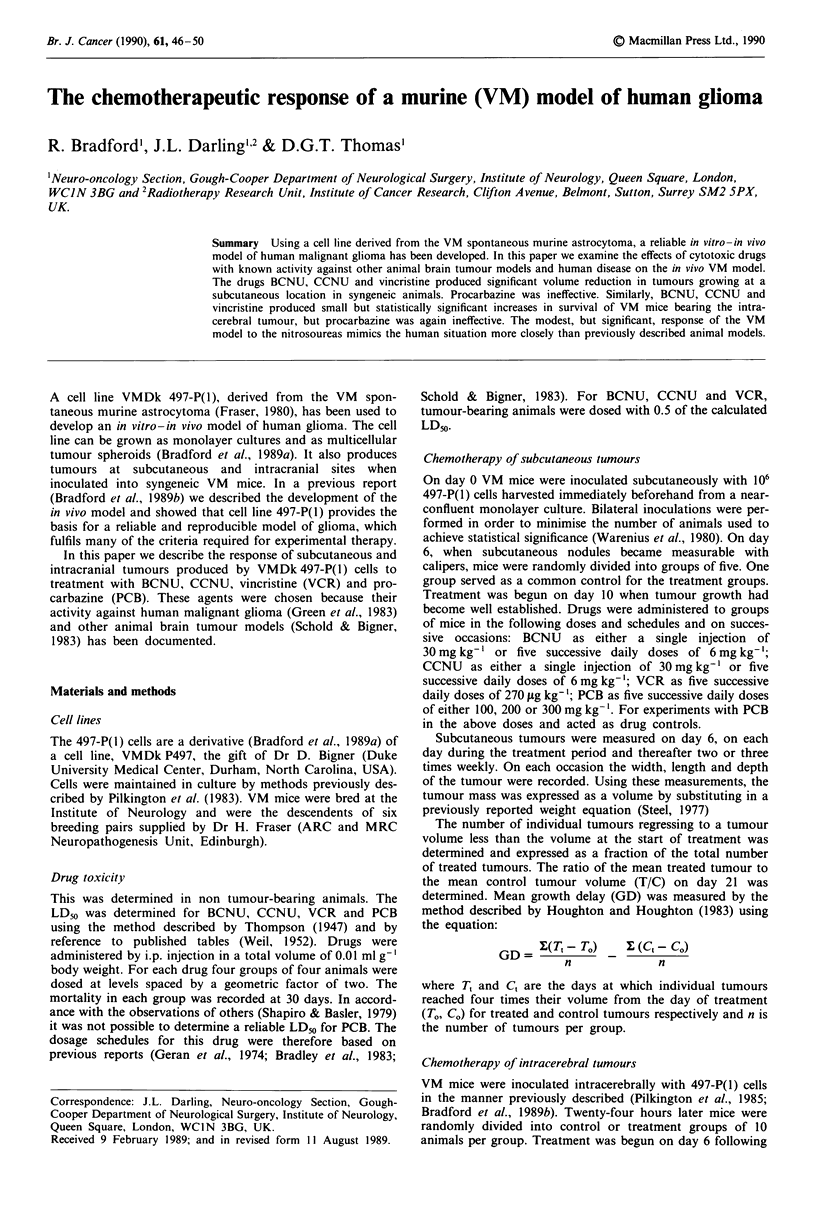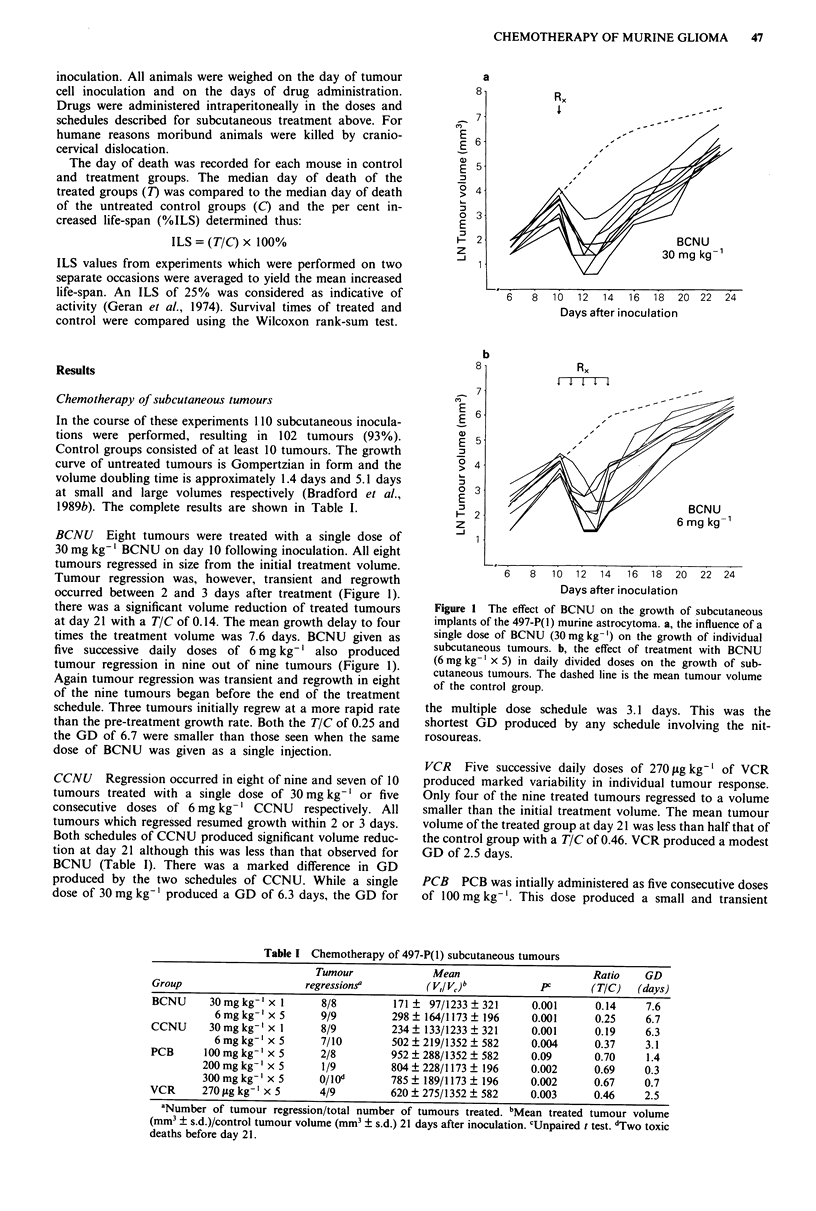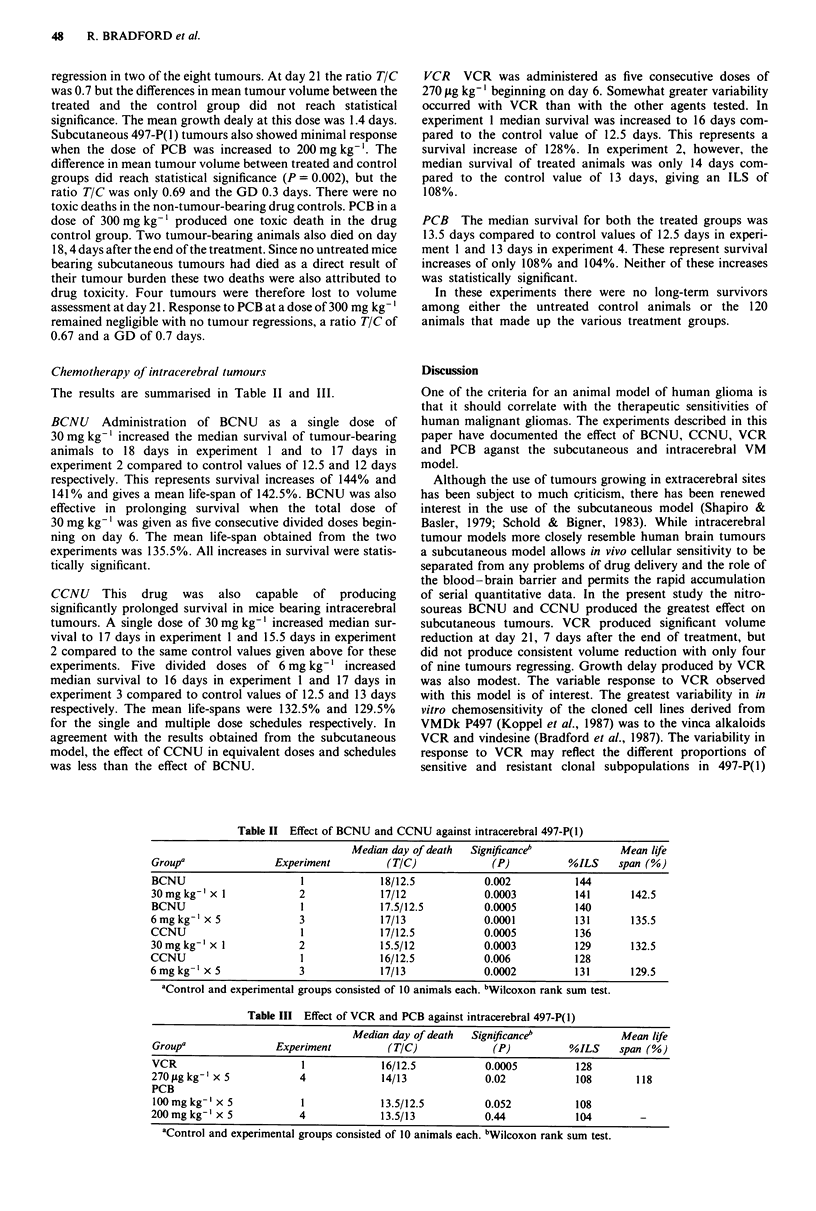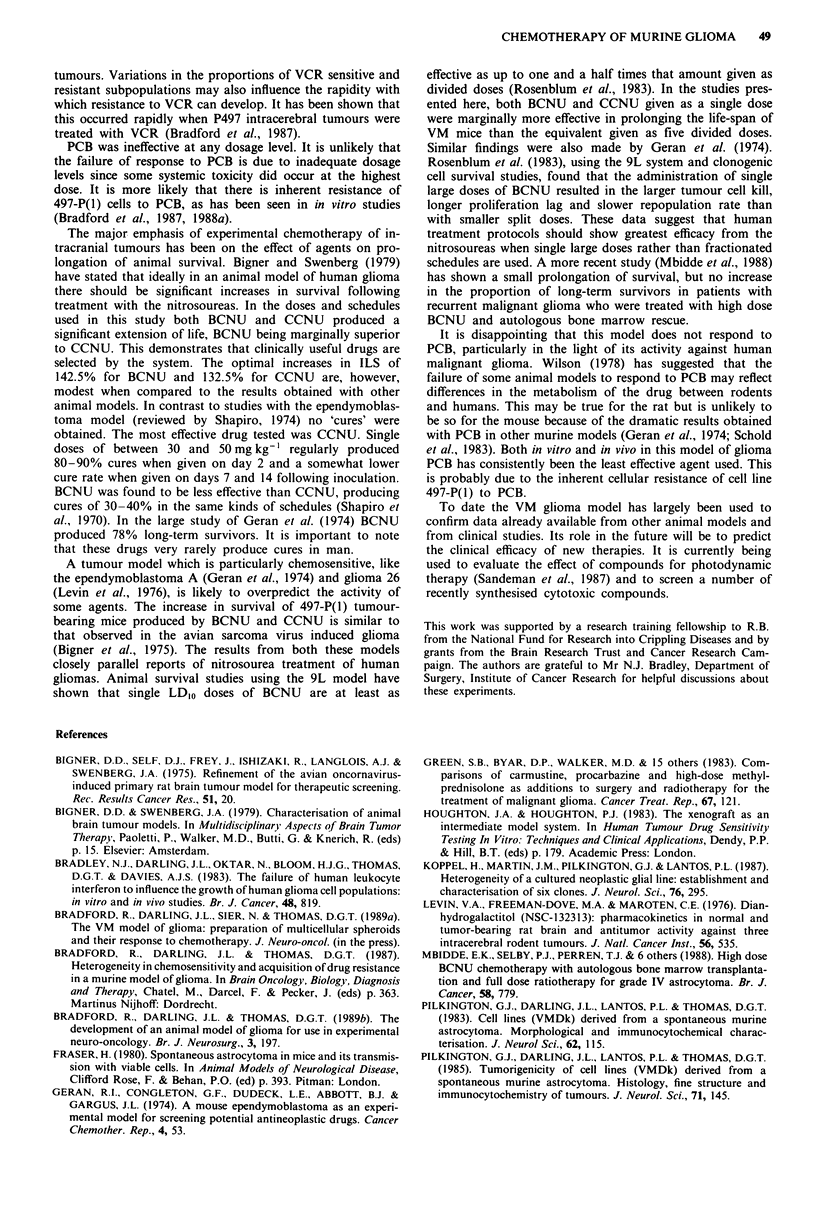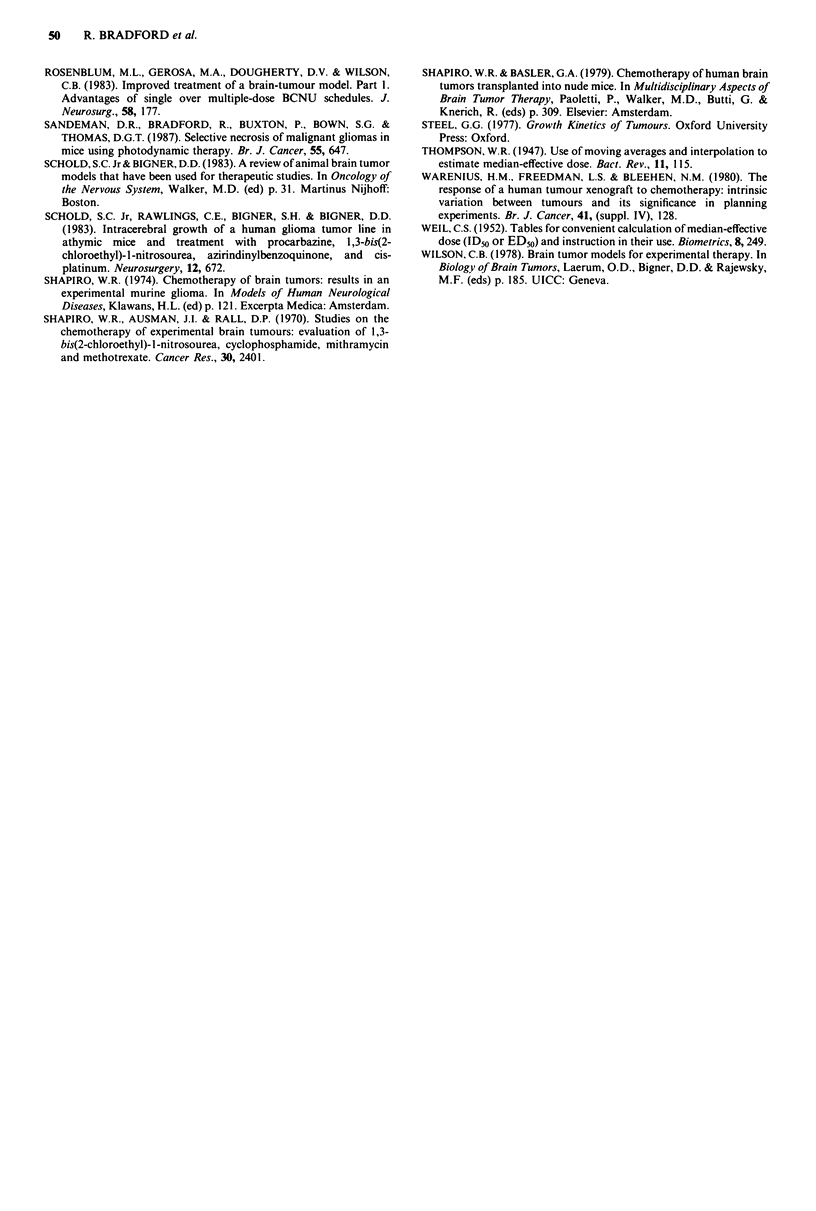# The chemotherapeutic response of a murine (VM) model of human glioma.

**DOI:** 10.1038/bjc.1990.10

**Published:** 1990-01

**Authors:** R. Bradford, J. L. Darling, D. G. Thomas

**Affiliations:** Gough-Cooper Department of Neurological Surgery, Institute of Neurology, London, UK.

## Abstract

Using a cell line derived from the VM spontaneous murine astrocytoma, a reliable in vitro-in vivo model of human malignant glioma has been developed. In this paper we examine the effects of cytotoxic drugs with known activity against other animal brain tumour models and human disease on the in vivo VM model. The drugs BCNU, CCNU and vincristine produced significant volume reduction in tumours growing at a subcutaneous location in syngeneic animals. Procarbazine was ineffective. Similarly, BCNU, CCNU and vincristine produced small but statistically significant increases in survival of VM mice bearing the intracerebral tumour, but procarbazine was again ineffective. The modest, but significant, response of the VM model to the nitrosoureas mimics the human situation more closely than previously described animal models.


Br. J. Cancer (1990), 61, 46–50 © Macmillan Press Ltd., 1990

### The chemotherapeutic response of a murine (VM) model of human glioma

R. Bradford[1], J.L. Darling[1,2] & D.G.T. Thomas[1]

[1]*Neuro-oncology Section, Gough-Cooper Department of Neurological Surgery, Institute of Neurology, Queen Square, London, WC1N 3BG and* [2]*Radiotherapy Research Unit, Institute of Cancer Research, Clifton Avenue, Belmont, Sutton, Surrey SM2 5PX, UK.*

**Summary** Using a cell line derived from the VM spontaneous murine astrocytoma, a reliable *in vitro–in vivo* model of human malignant glioma has been developed. In this paper we examine the effects of cytotoxic drugs with known activity against other animal brain tumour models and human disease on the *in vivo* VM model. The drugs BCNU, CCNU and vincristine produced significant volume reduction in tumours growing at a subcutaneous location in syngeneic animals. Procarbazine was ineffective. Similarly, BCNU, CCNU and vincristine produced small but statistically significant increases in survival of VM mice bearing the intracerebral tumour, but procarbazine was again ineffective. The modest, but significant, response of the VM model to the nitrosoureas mimics the human situation more closely than previously described animal models.

A cell line VMDk 497-P(1), derived from the VM spontaneous murine astrocytoma (Fraser, 1980), has been used to develop an *in vitro–in vivo* model of human glioma. The cell line can be grown as monolayer cultures and as multicellular tumour spheroids (Bradford *et al.*, 1989*a*). It also produces tumours at subcutaneous and intracranial sites when inoculated into syngeneic VM mice. In a previous report (Bradford *et al.*, 1989*b*) we described the development of the *in vivo* model and showed that cell line 497-P(1) provides the basis for a reliable and reproducible model of glioma, which fulfils many of the criteria required for experimental therapy.

In this paper we describe the response of subcutaneous and intracranial tumours produced by VMDk 497-P(1) cells to treatment with BCNU, CCNU, vincristine (VCR) and procarbazine (PCB). These agents were chosen because their activity against human malignant glioma (Green *et al.*, 1983) and other animal brain tumour models (Schold & Bigner, 1983) has been documented.

### Materials and methods

### *Cell lines*

The 497-P(1) cells are a derivative (Bradford *et al.*, 1989*a*) of a cell line, VMDk P497, the gift of Dr D. Bigner (Duke University Medical Center, Durham, North Carolina, USA). Cells were maintained in culture by methods previously described by Pilkington *et al.* (1983). VM mice were bred at the Institute of Neurology and were the descendents of six breeding pairs supplied by Dr H. Fraser (ARC and MRC Neuropathogenesis Unit, Edinburgh).

### *Drug toxicity*

This was determined in non tumour-bearing animals. The $LD_{50}$ was determined for BCNU, CCNU, VCR and PCB using the method described by Thompson (1947) and by reference to published tables (Weil, 1952). Drugs were administered by i.p. injection in a total volume of 0.01 ml $g^{-1}$ body weight. For each drug four groups of four animals were dosed at levels spaced by a geometric factor of two. The mortality in each group was recorded at 30 days. In accordance with the observations of others (Shapiro & Basler, 1979) it was not possible to determine a reliable $LD_{50}$ for PCB. The dosage schedules for this drug were therefore based on previous reports (Geran *et al.*, 1974; Bradley *et al.*, 1983; Schold & Bigner, 1983). For BCNU, CCNU and VCR, tumour-bearing animals were dosed with 0.5 of the calculated $LD_{50}$.

### *Chemotherapy of subcutaneous tumours*

On day 0 VM mice were inoculated subcutaneously with $10^6$ 497-P(1) cells harvested immediately beforehand from a near-confluent monolayer culture. Bilateral inoculations were performed in order to minimise the number of animals used to achieve statistical significance (Warenius *et al.*, 1980). On day 6, when subcutaneous nodules became measurable with calipers, mice were randomly divided into groups of five. One group served as a common control for the treatment groups. Treatment was begun on day 10 when tumour growth had become well established. Drugs were administered to groups of mice in the following doses and schedules and on successive occasions: BCNU as either a single injection of 30 mg $kg^{-1}$ or five successive daily doses of 6 mg $kg^{-1}$; CCNU as either a single injection of 30 mg $kg^{-1}$ or five successive daily doses of 6 mg $kg^{-1}$; VCR as five successive daily doses of 270 μg $kg^{-1}$; PCB as five successive daily doses of either 100, 200 or 300 mg $kg^{-1}$. For experiments with PCB in the above doses and acted as drug controls.

Subcutaneous tumours were measured on day 6, on each day during the treatment period and thereafter two or three times weekly. On each occasion the width, length and depth of the tumour were recorded. Using these measurements, the tumour mass was expressed as a volume by substituting in a previously reported weight equation (Steel, 1977)

The number of individual tumours regressing to a tumour volume less than the volume at the start of treatment was determined and expressed as a fraction of the total number of treated tumours. The ratio of the mean treated tumour to the mean control tumour volume (T/C) on day 21 was determined. Mean growth delay (GD) was measured by the method described by Houghton and Houghton (1983) using the equation:

$$GD = \frac{\Sigma(T_t - T_o)}{n} - \frac{\Sigma(C_t - C_o)}{n}$$

where $T_t$ and $C_t$ are the days at which individual tumours reached four times their volume from the day of treatment ($T_o$, $C_o$) for treated and control tumours respectively and $n$ is the number of tumours per group.

### *Chemotherapy of intracerebral tumours*

VM mice were inoculated intracerebrally with 497-P(1) cells in the manner previously described (Pilkington *et al.*, 1985; Bradford *et al.*, 1989*b*). Twenty-four hours later mice were randomly divided into control or treatment groups of 10 animals per group. Treatment was begun on day 6 following

Correspondence: J.L. Darling, Neuro-oncology Section, Gough-Cooper Department of Neurological Surgery, Institute of Neurology, Queen Square, London, WC1N 3BG, UK.
Received 9 February 1989; and in revised form 11 August 1989.

inoculation. All animals were weighed on the day of tumour cell inoculation and on the days of drug administration. Drugs were administered intraperitoneally in the doses and schedules described for subcutaneous treatment above. For humane reasons moribund animals were killed by craniocervical dislocation.

The day of death was recorded for each mouse in control and treatment groups. The median day of death of the treated groups ($T$) was compared to the median day of death of the untreated control groups ($C$) and the per cent increased life-span (%ILS) determined thus:

$$ILS = (T/C) \times 100\%$$

ILS values from experiments which were performed on two separate occasions were averaged to yield the mean increased life-span. An ILS of 25% was considered as indicative of activity (Geran *et al.*, 1974). Survival times of treated and control were compared using the Wilcoxon rank-sum test.

### Results

*Chemotherapy of subcutaneous tumours*

In the course of these experiments 110 subcutaneous inoculations were performed, resulting in 102 tumours (93%). Control groups consisted of at least 10 tumours. The growth curve of untreated tumours is Gompertzian in form and the volume doubling time is approximately 1.4 days and 5.1 days at small and large volumes respectively (Bradford *et al.*, 1989*b*). The complete results are shown in Table I.

*BCNU* Eight tumours were treated with a single dose of 30 mg kg$^{-1}$ BCNU on day 10 following inoculation. All eight tumours regressed in size from the initial treatment volume. Tumour regression was, however, transient and regrowth occurred between 2 and 3 days after treatment (Figure 1). there was a significant volume reduction of treated tumours at day 21 with a $T/C$ of 0.14. The mean growth delay to four times the treatment volume was 7.6 days. BCNU given as five successive daily doses of 6 mg kg$^{-1}$ also produced tumour regression in nine out of nine tumours (Figure 1). Again tumour regression was transient and regrowth in eight of the nine tumours began before the end of the treatment schedule. Three tumours initially regrew at a more rapid rate than the pre-treatment growth rate. Both the $T/C$ of 0.25 and the GD of 6.7 were smaller than those seen when the same dose of BCNU was given as a single injection.

*CCNU* Regression occurred in eight of nine and seven of 10 tumours treated with a single dose of 30 mg kg$^{-1}$ or five consecutive doses of 6 mg kg$^{-1}$ CCNU respectively. All tumours which regressed resumed growth within 2 or 3 days. Both schedules of CCNU produced significant volume reduction at day 21 although this was less than that observed for BCNU (Table I). There was a marked difference in GD produced by the two schedules of CCNU. While a single dose of 30 mg kg$^{-1}$ produced a GD of 6.3 days, the GD for

**Figure 1** The effect of BCNU on the growth of subcutaneous implants of the 497-P(1) murine astrocytoma. **a**, the influence of a single dose of BCNU (30 mg kg$^{-1}$) on the growth of individual subcutaneous tumours. **b**, the effect of treatment with BCNU (6 mg kg$^{-1}$ × 5) in daily divided doses on the growth of subcutaneous tumours. The dashed line is the mean tumour volume of the control group.

the multiple dose schedule was 3.1 days. This was the shortest GD produced by any schedule involving the nitrosoureas.

*VCR* Five successive daily doses of 270 µg kg$^{-1}$ of VCR produced marked variability in individual tumour response. Only four of the nine treated tumours regressed to a volume smaller than the initial treatment volume. The mean tumour volume of the treated group at day 21 was less than half that of the control group with a $T/C$ of 0.46. VCR produced a modest GD of 2.5 days.

*PCB* PCB was intially administered as five consecutive doses of 100 mg kg$^{-1}$. This dose produced a small and transient

**Table I** Chemotherapy of 497-P(1) subcutaneous tumours

| Group | | *Tumour regressions*[a] | *Mean* $(V_t/V_c)^b$ | $P^c$ | *Ratio* $(T/C)$ | *GD (days)* |
|---|---|---|---|---|---|---|
| BCNU | 30 mg kg$^{-1}$ × 1 | 8/8 | 171 ± 97/1233 ± 321 | 0.001 | 0.14 | 7.6 |
| | 6 mg kg$^{-1}$ × 5 | 9/9 | 298 ± 164/1173 ± 196 | 0.001 | 0.25 | 6.7 |
| CCNU | 30 mg kg$^{-1}$ × 1 | 8/9 | 234 ± 133/1233 ± 321 | 0.001 | 0.19 | 6.3 |
| | 6 mg kg$^{-1}$ × 5 | 7/10 | 502 ± 219/1352 ± 582 | 0.004 | 0.37 | 3.1 |
| PCB | 100 mg kg$^{-1}$ × 5 | 2/8 | 952 ± 288/1352 ± 582 | 0.09 | 0.70 | 1.4 |
| | 200 mg kg$^{-1}$ × 5 | 1/9 | 804 ± 228/1173 ± 196 | 0.002 | 0.69 | 0.3 |
| | 300 mg kg$^{-1}$ × 5 | 0/10[d] | 785 ± 189/1173 ± 196 | 0.002 | 0.67 | 0.7 |
| VCR | 270 µg kg$^{-1}$ × 5 | 4/9 | 620 ± 275/1352 ± 582 | 0.003 | 0.46 | 2.5 |

[a]Number of tumour regression/total number of tumours treated. [b]Mean treated tumour volume (mm$^3$ ± s.d.)/control tumour volume (mm$^3$ ± s.d.) 21 days after inoculation. [c]Unpaired $t$ test. [d]Two toxic deaths before day 21.

regression in two of the eight tumours. At day 21 the ratio *T/C* was 0.7 but the differences in mean tumour volume between the treated and the control group did not reach statistical significance. The mean growth dealy at this dose was 1.4 days. Subcutaneous 497-P(1) tumours also showed minimal response when the dose of PCB was increased to 200 mg $kg^{-1}$. The difference in mean tumour volume between treated and control groups did reach statistical significance ($P = 0.002$), but the ratio *T/C* was only 0.69 and the GD 0.3 days. There were no toxic deaths in the non-tumour-bearing drug controls. PCB in a dose of 300 mg $kg^{-1}$ produced one toxic death in the drug control group. Two tumour-bearing animals also died on day 18, 4 days after the end of the treatment. Since no untreated mice bearing subcutaneous tumours had died as a direct result of their tumour burden these two deaths were also attributed to drug toxicity. Four tumours were therefore lost to volume assessment at day 21. Response to PCB at a dose of 300 mg $kg^{-1}$ remained negligible with no tumour regressions, a ratio *T/C* of 0.67 and a GD of 0.7 days.

*Chemotherapy of intracerebral tumours*

The results are summarised in Table II and III.

*BCNU* Administration of BCNU as a single dose of 30 mg $kg^{-1}$ increased the median survival of tumour-bearing animals to 18 days in experiment 1 and to 17 days in experiment 2 compared to control values of 12.5 and 12 days respectively. This represents survival increases of 144% and 141% and gives a mean life-span of 142.5%. BCNU was also effective in prolonging survival when the total dose of 30 mg $kg^{-1}$ was given as five consecutive divided doses beginning on day 6. The mean life-span obtained from the two experiments was 135.5%. All increases in survival were statistically significant.

*CCNU* This drug was also capable of producing significantly prolonged survival in mice bearing intracerebral tumours. A single dose of 30 mg $kg^{-1}$ increased median survival to 17 days in experiment 1 and 15.5 days in experiment 2 compared to the same control values given above for these experiments. Five divided doses of 6 mg $kg^{-1}$ increased median survival to 16 days in experiment 1 and 17 days in experiment 3 compared to control values of 12.5 and 13 days respectively. The mean life-spans were 132.5% and 129.5% for the single and multiple dose schedules respectively. In agreement with the results obtained from the subcutaneous model, the effect of CCNU in equivalent doses and schedules was less than the effect of BCNU.

*VCR* VCR was administered as five consecutive doses of 270 μg $kg^{-1}$ beginning on day 6. Somewhat greater variability occurred with VCR than with the other agents tested. In experiment 1 median survival was increased to 16 days compared to the control value of 12.5 days. This represents a survival increase of 128%. In experiment 2, however, the median survival of treated animals was only 14 days compared to the control value of 13 days, giving an ILS of 108%.

*PCB* The median survival for both the treated groups was 13.5 days compared to control values of 12.5 days in experiment 1 and 13 days in experiment 4. These represent survival increases of only 108% and 104%. Neither of these increases was statistically significant.

In these experiments there were no long-term survivors among either the untreated control animals or the 120 animals that made up the various treatment groups.

### Discussion

One of the criteria for an animal model of human glioma is that it should correlate with the therapeutic sensitivities of human malignant gliomas. The experiments described in this paper have documented the effect of BCNU, CCNU, VCR and PCB aganst the subcutaneous and intracerebral VM model.

Although the use of tumours growing in extracerebral sites has been subject to much criticism, there has been renewed interest in the use of the subcutaneous model (Shapiro & Basler, 1979; Schold & Bigner, 1983). While intracerebral tumour models more closely resemble human brain tumours a subcutaneous model allows *in vivo* cellular sensitivity to be separated from any problems of drug delivery and the role of the blood–brain barrier and permits the rapid accumulation of serial quantitative data. In the present study the nitrosoureas BCNU and CCNU produced the greatest effect on subcutaneous tumours. VCR produced significant volume reduction at day 21, 7 days after the end of treatment, but did not produce consistent volume reduction with only four of nine tumours regressing. Growth delay produced by VCR was also modest. The variable response to VCR observed with this model is of interest. The greatest variability in *in vitro* chemosensitivity of the cloned cell lines derived from VMDk P497 (Koppel *et al.*, 1987) was to the vinca alkaloids VCR and vindesine (Bradford *et al.*, 1987). The variability in response to VCR may reflect the different proportions of sensitive and resistant clonal subpopulations in 497-P(1)

**Table II** Effect of BCNU and CCNU against intracerebral 497-P(1)

| *Group*[a] | *Experiment* | *Median day of death (T/C)* | *Significance*[b] *(P)* | *%ILS* | *Mean life span (%)* |
|---|---|---|---|---|---|
| BCNU | 1 | 18/12.5 | 0.002 | 144 | |
| 30 mg $kg^{-1}$ × 1 | 2 | 17/12 | 0.0003 | 141 | 142.5 |
| BCNU | 1 | 17.5/12.5 | 0.0005 | 140 | |
| 6 mg $kg^{-1}$ × 5 | 3 | 17/13 | 0.0001 | 131 | 135.5 |
| CCNU | 1 | 17/12.5 | 0.0005 | 136 | |
| 30 mg $kg^{-1}$ × 1 | 2 | 15.5/12 | 0.0003 | 129 | 132.5 |
| CCNU | 1 | 16/12.5 | 0.006 | 128 | |
| 6 mg $kg^{-1}$ × 5 | 3 | 17/13 | 0.0002 | 131 | 129.5 |

[a]Control and experimental groups consisted of 10 animals each. [b]Wilcoxon rank sum test.

**Table III** Effect of VCR and PCB against intracerebral 497-P(1)

| *Group*[a] | *Experiment* | *Median day of death (T/C)* | *Significance*[b] *(P)* | *%ILS* | *Mean life span (%)* |
|---|---|---|---|---|---|
| VCR | 1 | 16/12.5 | 0.0005 | 128 | |
| 270 μg $kg^{-1}$ × 5 | 4 | 14/13 | 0.02 | 108 | 118 |
| PCB | | | | | |
| 100 mg $kg^{-1}$ × 5 | 1 | 13.5/12.5 | 0.052 | 108 | |
| 200 mg $kg^{-1}$ × 5 | 4 | 13.5/13 | 0.44 | 104 | – |

[a]Control and experimental groups consisted of 10 animals each. [b]Wilcoxon rank sum test.

tumours. Variations in the proportions of VCR sensitive and resistant subpopulations may also influence the rapidity with which resistance to VCR can develop. It has been shown that this occurred rapidly when P497 intracerebral tumours were treated with VCR (Bradford *et al.*, 1987).

PCB was ineffective at any dosage level. It is unlikely that the failure of response to PCB is due to inadequate dosage levels since some systemic toxicity did occur at the highest dose. It is more likely that there is inherent resistance of 497-P(1) cells to PCB, as has been seen in *in vitro* studies (Bradford *et al.*, 1987, 1988*a*).

The major emphasis of experimental chemotherapy of intracranial tumours has been on the effect of agents on prolongation of animal survival. Bigner and Swenberg (1979) have stated that ideally in an animal model of human glioma there should be significant increases in survival following treatment with the nitrosoureas. In the doses and schedules used in this study both BCNU and CCNU produced a significant extension of life, BCNU being marginally superior to CCNU. This demonstrates that clinically useful drugs are selected by the system. The optimal increases in ILS of 142.5% for BCNU and 132.5% for CCNU are, however, modest when compared to the results obtained with other animal models. In contrast to studies with the ependymoblastoma model (reviewed by Shapiro, 1974) no 'cures' were obtained. The most effective drug tested was CCNU. Single doses of between 30 and 50 mg $kg^{-1}$ regularly produced 80–90% cures when given on day 2 and a somewhat lower cure rate when given on days 7 and 14 following inoculation. BCNU was found to be less effective than CCNU, producing cures of 30–40% in the same kinds of schedules (Shapiro *et al.*, 1970). In the large study of Geran *et al.* (1974) BCNU produced 78% long-term survivors. It is important to note that these drugs very rarely produce cures in man.

A tumour model which is particularly chemosensitive, like the ependymoblastoma A (Geran *et al.*, 1974) and glioma 26 (Levin *et al.*, 1976), is likely to overpredict the activity of some agents. The increase in survival of 497-P(1) tumour-bearing mice produced by BCNU and CCNU is similar to that observed in the avian sarcoma virus induced glioma (Bigner *et al.*, 1975). The results from both these models closely parallel reports of nitrosourea treatment of human gliomas. Animal survival studies using the 9L model have shown that single $LD_{10}$ doses of BCNU are at least as effective as up to one and a half times that amount given as divided doses (Rosenblum *et al.*, 1983). In the studies presented here, both BCNU and CCNU given as a single dose were marginally more effective in prolonging the life-span of VM mice than the equivalent given as five divided doses. Similar findings were also made by Geran *et al.* (1974). Rosenblum *et al.* (1983), using the 9L system and clonogenic cell survival studies, found that the administration of single large doses of BCNU resulted in the larger tumour cell kill, longer proliferation lag and slower repopulation rate than with smaller split doses. These data suggest that human treatment protocols should show greatest efficacy from the nitrosoureas when single large doses rather than fractionated schedules are used. A more recent study (Mbidde *et al.*, 1988) has shown a small prolongation of survival, but no increase in the proportion of long-term survivors in patients with recurrent malignant glioma who were treated with high dose BCNU and autologous bone marrow rescue.

It is disappointing that this model does not respond to PCB, particularly in the light of its activity against human malignant glioma. Wilson (1978) has suggested that the failure of some animal models to respond to PCB may reflect differences in the metabolism of the drug between rodents and humans. This may be true for the rat but is unlikely to be so for the mouse because of the dramatic results obtained with PCB in other murine models (Geran *et al.*, 1974; Schold *et al.*, 1983). Both *in vitro* and *in vivo* in this model of glioma PCB has consistently been the least effective agent used. This is probably due to the inherent cellular resistance of cell line 497-P(1) to PCB.

To date the VM glioma model has largely been used to confirm data already available from other animal models and from clinical studies. Its role in the future will be to predict the clinical efficacy of new therapies. It is currently being used to evaluate the effect of compounds for photodynamic therapy (Sandeman *et al.*, 1987) and to screen a number of recently synthesised cytotoxic compounds.

This work was supported by a research training fellowship to R.B. from the National Fund for Research into Crippling Diseases and by grants from the Brain Research Trust and Cancer Research Campaign. The authors are grateful to Mr N.J. Bradley, Department of Surgery, Institute of Cancer Research for helpful discussions about these experiments.

### References

BIGNER, D.D., SELF, D.J., FREY, J., ISHIZAKI, R., LANGLOIS, A.J. & SWENBERG, J.A. (1975). Refinement of the avian oncornavirus-induced primary rat brain tumour model for therapeutic screening. *Rec. Results Cancer Res.*, **51**, 20.

BIGNER, D.D. & SWENBERG, J.A. (1979). Characterisation of animal brain tumour models. In *Multidisciplinary Aspects of Brain Tumor Therapy*, Paoletti, P., Walker, M.D., Butti, G. & Knerich, R. (eds) p. 15. Elsevier: Amsterdam.

BRADLEY, N.J., DARLING, J.L., OKTAR, N., BLOOM, H.J.G., THOMAS, D.G.T. & DAVIES, A.J.S. (1983). The failure of human leukocyte interferon to influence the growth of human glioma cell populations: *in vitro* and *in vivo* studies. *Br. J. Cancer*, **48**, 819.

BRADFORD, R., DARLING, J.L., SIER, N. & THOMAS, D.G.T. (1989*a*). The VM model of glioma: preparation of multicellular spheroids and their response to chemotherapy. *J. Neuro-oncol.* (in the press).

BRADFORD, R., DARLING, J.L. & THOMAS, D.G.T. (1987). Heterogeneity in chemosensitivity and acquisition of drug resistance in a murine model of glioma. In *Brain Oncology, Biology, Diagnosis and Therapy*, Chatel, M., Darcel, F. & Pecker, J. (eds) p. 363. Martinus Nijhoff: Dordrecht.

BRADFORD, R., DARLING, J.L. & THOMAS, D.G.T. (1989*b*). The development of an animal model of glioma for use in experimental neuro-oncology. *Br. J. Neurosurg.*, **3**, 197.

FRASER, H. (1980). Spontaneous astrocytoma in mice and its transmission with viable cells. In *Animal Models of Neurological Disease*, Clifford Rose, F. & Behan, P.O. (ed) p. 393. Pitman: London.

GERAN, R.I., CONGLETON, G.F., DUDECK, L.E., ABBOTT, B.J. & GARGUS, J.L. (1974). A mouse ependymoblastoma as an experimental model for screening potential antineoplastic drugs. *Cancer Chemother. Rep.*, **4**, 53.

GREEN, S.B., BYAR, D.P., WALKER, M.D. & 15 others (1983). Comparisons of carmustine, procarbazine and high-dose methylprednisolone as additions to surgery and radiotherapy for the treatment of malignant glioma. *Cancer Treat. Rep.*, **67**, 121.

HOUGHTON, J.A. & HOUGHTON, P.J. (1983). The xenograft as an intermediate model system. In *Human Tumour Drug Sensitivity Testing In Vitro: Techniques and Clinical Applications*, Dendy, P.P. & Hill, B.T. (eds) p. 179. Academic Press: London.

KOPPEL, H., MARTIN, J.M., PILKINGTON, G.J. & LANTOS, P.L. (1987). Heterogeneity of a cultured neoplastic glial line: establishment and characterisation of six clones. *J. Neurol. Sci.*, **76**, 295.

LEVIN, V.A., FREEMAN-DOVE, M.A. & MAROTEN, C.E. (1976). Dianhydrogalactitol (NSC-132313): pharmacokinetics in normal and tumor-bearing rat brain and antitumor activity against three intracerebral rodent tumours. *J. Natl. Cancer Inst.*, **56**, 535.

MBIDDE, E.K., SELBY, P.J., PERREN, T.J. & 6 others (1988). High dose BCNU chemotherapy with autologous bone marrow transplantation and full dose ratiotherapy for grade IV astrocytoma. *Br. J. Cancer*, **58**, 779.

PILKINGTON, G.J., DARLING, J.L., LANTOS, P.L. & THOMAS, D.G.T. (1983). Cell lines (VMDk) derived from a spontaneous murine astrocytoma. Morphological and immunocytochemical characterisation. *J. Neurol Sci.*, **62**, 115.

PILKINGTON, G.J., DARLING, J.L., LANTOS, P.L. & THOMAS, D.G.T. (1985). Tumorigenicity of cell lines (VMDk) derived from a spontaneous murine astrocytoma. Histology, fine structure and immunocytochemistry of tumours. *J. Neurol. Sci.*, **71**, 145.

ROSENBLUM, M.L., GEROSA, M.A., DOUGHERTY, D.V. & WILSON, C.B. (1983). Improved treatment of a brain-tumour model. Part 1. Advantages of single over multiple-dose BCNU schedules. *J. Neurosurg.*, **58,** 177.

SANDEMAN, D.R., BRADFORD, R., BUXTON, P., BOWN, S.G. & THOMAS, D.G.T. (1987). Selective necrosis of malignant gliomas in mice using photodynamic therapy. *Br. J. Cancer*, **55,** 647.

SCHOLD, S.C. Jr & BIGNER, D.D. (1983). A review of animal brain tumor models that have been used for therapeutic studies. In *Oncology of the Nervous System*, Walker, M.D. (ed) p. 31. Martinus Nijhoff: Boston.

SCHOLD, S.C. Jr, RAWLINGS, C.E., BIGNER, S.H. & BIGNER, D.D. (1983). Intracerebral growth of a human glioma tumor line in athymic mice and treatment with procarbazine, 1,3-*bis*(2-chloroethyl)-1-nitrosourea, azirindinylbenzoquinone, and cis-platinum. *Neurosurgery*, **12,** 672.

SHAPIRO, W.R. (1974). Chemotherapy of brain tumors: results in an experimental murine glioma. In *Models of Human Neurological Diseases*, Klawans, H.L. (ed) p. 121. Excerpta Medica: Amsterdam.

SHAPIRO, W.R., AUSMAN, J.I. & RALL, D.P. (1970). Studies on the chemotherapy of experimental brain tumours: evaluation of 1,3-*bis*(2-chloroethyl)-1-nitrosourea, cyclophosphamide, mithramycin and methotrexate. *Cancer Res.*, **30,** 2401.

SHAPIRO, W.R. & BASLER, G.A. (1979). Chemotherapy of human brain tumors transplanted into nude mice. In *Multidisciplinary Aspects of Brain Tumor Therapy*, Paoletti, P., Walker, M.D., Butti, G. & Knerich, R. (eds) p. 309. Elsevier: Amsterdam.

STEEL, G.G. (1977). *Growth Kinetics of Tumours*. Oxford University Press: Oxford.

THOMPSON, W.R. (1947). Use of moving averages and interpolation to estimate median-effective dose. *Bact. Rev.*, **11,** 115.

WARENIUS, H.M., FREEDMAN, L.S. & BLEEHEN, N.M. (1980). The response of a human tumour xenograft to chemotherapy: intrinsic variation between tumours and its significance in planning experiments. *Br. J. Cancer*, **41,** (suppl. IV), 128.

WEIL, C.S. (1952). Tables for convenient calculation of median-effective dose ($ID_{50}$ or $ED_{50}$) and instruction in their use. *Biometrics*, **8,** 249.

WILSON, C.B. (1978). Brain tumor models for experimental therapy. In *Biology of Brain Tumors*, Laerum, O.D., Bigner, D.D. & Rajewsky, M.F. (eds) p. 185. UICC: Geneva.